# Treatment Preferences for Pharmacological versus Psychological Interventions among Primary Care Providers in Nepal: Mixed Methods Analysis of a Pilot Cluster Randomized Controlled Trial

**DOI:** 10.3390/ijerph19042149

**Published:** 2022-02-14

**Authors:** Anvita Bhardwaj, Dristy Gurung, Sauharda Rai, Bonnie N. Kaiser, Cori L. Cafaro, Kathleen J. Sikkema, Crick Lund, Nagendra P. Luitel, Brandon A. Kohrt

**Affiliations:** 1 Department of Mental Health, Johns Hopkins Bloomberg School of Public Health, Baltimore, MD 21205, USA; abhardwaj@jhu.edu; 2 Duke Global Health Institute, Duke University, Durham, NC 27710, USA; sauharda@uw.edu (S.R.); bnkaiser@ucsd.edu (B.N.K.); ctergese@depaul.edu (C.L.C.); ks3364@cumc.columbia.edu (K.J.S.); 3 Centre for Global Mental Health, Health Services and Population Research Department, Institute of Psychiatry, Psychology and Neuroscience, King’s College London, London SE5 8AF, UK; dristy.1.gurung@kcl.ac.uk (D.G.); crick.lund@kcl.ac.uk (C.L.); 4 Transcultural Psychosocial Organization (TPO) Nepal, Baluwatar 44616, Nepal; luitelnp@gmail.com; 5 Jackson School of International Studies and Department of Global Health, University of Washington, Seattle, WA 98195, USA; 6 Department of Anthropology and Global Health Program, University of California San Diego, La Jolla, CA 92093, USA; 7 Department of Psychology, DePaul University, Chicago, IL 60604, USA; 8 Department of Sociomedical Sciences, Mailman School of Public Health, Columbia University, New York, NY 10032, USA; 9 Global Mental Health and Development, King’s Global Health Institute, King’s College London, London WC2R 2LS, UK; 10 Alan J. Flisher Centre for Public Mental Health, Department of Psychiatry and Mental Health, University of Cape Town, Cape Town 7700, South Africa; 11 Department of Psychiatry and Behavioral Sciences, The George Washington University, Washington, DC 20037, USA

**Keywords:** attitudes, depression, developing countries, mental health, primary care, psychological treatments, stigma, training

## Abstract

There is increasing evidence supporting the effectiveness of psychological interventions in low- and middle-income countries. However, primary care providers (PCPs) may prefer treating patients with medication. A secondary exploratory analysis of a pilot cluster randomized controlled trial was conducted to evaluate psychological vs. pharmacological treatment preferences among PCPs. Thirty-four health facilities, including 205 PCPs, participated in the study, with PCPs in 17 facilities assigned to a standard version of the mental health Gap Action Programme (mhGAP) training delivered by mental health specialists. PCPs in the other 17 facilities received mhGAP instruction delivered by specialists and people with lived experience of mental illness (PWLE), using a training strategy entitled Reducing Stigma among HealthcAre ProvidErs (RESHAPE). Pre- and post- intervention attitudes were measured through quantitative and qualitative tools. Qualitative interviews with 49 participants revealed that PCPs in both arms endorsed counseling’s benefits and collaboration within the health system to provide counseling. In the RESHAPE arm, PCPs were more likely to increase endorsement of statements such as “depression improves without medication” (*F* = 9.83, *p* < 0.001), “not all people with depression must be treated with antidepressants” (*χ*^2^ = 17.62, *p* < 0.001), and “providing counseling to people who have alcohol abuse problems is effective” (*χ*^2^ = 26.20, *p* < 0.001). These mixed-method secondary findings from a pilot trial suggest that in-person participation of PWLE in training PCPs may not only reduce stigma but also increase PCPs’ support of psychological interventions. This requires further investigation in a full-scale trial.

## 1. Introduction

When addressing the global treatment gap for those suffering from mental, neurological, and substance use disorders, it is important to ensure that primary care providers (PCPs) utilize evidence-based treatments [1]. Psychological interventions are evidence-based treatments that can be effectively delivered by people who are not mental health specialists, including providers in primary care and community care in low-resource settings [2]. Training initiatives such as the World Health Organization (WHO) mental health Gap Action Programme-Intervention Guide (mhGAP-IG) encourage the use of both psychological and pharmacological treatments in primary care settings [3]. However, given that PCPs and patients in low- and middle-income countries (LMICs) are accustomed to medications as the standard treatments for non-psychiatric conditions, there is a risk that they favor the adoption of psychiatric medication to the neglect of psychotherapies [4]. For example, among Nigerian primary care physicians trained in mental health care, 88% endorsed that psychotherapy tended to be unsuccessful among patients with depression, and 85% endorsed that psychotherapy should be left to specialists [5]. In the same study, 85% supported prescribing anti-depressant medication by non-psychiatric health workers such as PCPs. This preference for medication may also come from expectations to see large numbers of patients in a limited time, which makes it challenging to integrate time-intensive psychological treatments into primary care [6]. Ethnographic accounts in South Asia and Sub-Saharan Africa also reveal a preference for pharmacological treatment by PCPs and patients, as well as by many mental health specialists [7,8,9]. Concerns have previously been raised in other settings that the mhGAP-IG curriculum may reinforce these biases in favor of pharmacological treatment and further contribute to the medicalization of care for mental illnesses [4].

Despite psychotropic medication being the default treatment in primary care settings in LMICs, there are a number of reasons for trying to ensure the availability and implementation of psychological interventions. Psychological interventions are as effective as medications for treatment of common mental disorders, such as depression [10]. Psychological therapies used in combination with medications lead to better outcomes than medication alone [11]. Moreover, LMIC settings are vulnerable to shortages of medications, especially psychotropic drugs, which makes sole reliance on pharmacological medications untenable. In Tanzania and India, psychotropic medications are on essential drug lists established by the WHO, but there are inadequate supplies in primary care facilities due to delays in procurement and lack of dispensing, which causes medications to expire [12,13]. In Nepal, a comprehensive array of up-to-date, safe, and effective psychotropic medications was only recently added to the essential drug list by the government, but the supply chain is not yet reliable [14]. The COVID-19 pandemic has further disrupted psychiatric medication supply chains in LMICs including Nepal, while the increased use of mobile technology has widened the opportunities for psychological interventions [15].

Research from high-income countries (HICs) suggests that when patients are familiar with psychological treatments, they are more likely to select these over medication when available [16]. A review of patients’ preferences based on 34 studies conducted in HICs demonstrated that 75% of patients preferred solely psychological treatments, and this preference was stronger among ethnic minorities, specifically in African Americans, Asian/Pacific Islanders, and Hispanic Americans in comparison to non-Hispanic white participants [16]. Overall, the current literature suggests that when patients are aware of psychological treatments, they prefer these, especially for conditions of mild to moderate severity. In LMICs, this requires that PCPs educate their patients about the availability and benefit of psychological treatments. Moreover, the availability of both psychological and pharmacological treatment is consistent with the Integrative Mental Health (IMH) paradigm. IMH aims to reconcile the biological, psychological, sociological, and spiritual models that play a role in mental health care, thus promoting the use of evidence-based psychological treatments alongside pharmacotherapies [17]. The IMH paradigm focuses on treating someone as a whole person, exploring causal factors and promoting education and empowerment of patients [17]. In global mental health, and in Nepal in particular, an integrative framework is needed to balance psychological, pharmacological, and non-allopathic care such as traditional healing [18,19,20,21].

The goal of this study was to evaluate the impact of mhGAP-IG training on treatment preferences among PCPs in a low-resource setting. We evaluated two strategies to implementing mhGAP-IG training for PCPs to determine if the endorsement of psychological treatments would potentially vary based on how training was delivered. Using a pilot cluster randomized controlled trial (RCT) design, we assigned primary care facilities to either standard mhGAP-IG training delivered by mental health specialists or an experimental arm in which mhGAP-IG training was delivered by mental health specialists supplemented with components of the training delivered by people with lived experience of mental illness (PWLE) and aspirational figures, who are PCPs that have taken on mental healthcare delivery [22]. This experimental training strategy is entitled Reducing Stigma among HealthcAre ProvidErs (RESHAPE). We hypothesized that the RESHAPE version of mhGAP-IG would contribute to greater endorsement of psychological treatment because of the empathy and humanistic perspective fostered through social contact with PWLE [23,24,25,26]. The pilot findings suggest that RESHAPE improves both attitudes towards patients with mental illness and accuracy of mental illness diagnoses [27]. In the current analysis, we conduct a secondary exploration of the trial data and use a mixed methods approach to evaluate quantitative differences in treatment attitudes of PCPs across arms. We also explore qualitative themes related to treatment preferences of the PCPs participating in the training.

## 2. Materials and Methods

### 2.1. Training Strategies for Primary Care Providers: Standard mhGAP vs. RESHAPE

This study was conducted within the context of the Programme for Improving Mental health carE (PRIME), which was an initiative to develop mental health services in primary care and community settings in LMICs using evidence-supported pharmacological and psychological treatment protocols including mhGAP-IG [28]. PRIME research was conducted in five countries, including in Nepal, where it was implemented through Transcultural Psychosocial Organization (TPO) Nepal, a non-governmental organization, in Chitwan district in the southern region of the country [29].

Mental health services were integrated within the primary health care system in Chitwan based on a district mental healthcare plan [30]. The plan relies upon psychiatrists and experienced psychosocial counselors as trainers and supervisors, with the majority of care delivered by government PCPs in accordance with mhGAP-IG and support from community health volunteers and community psychosocial counselors [30]. PCPs include health assistants, medical officers, and auxiliary health workers, who are termed *prescribers* due to their authority in the primary health facility to prescribe medicines. Another class of primary care staff comprises auxiliary nurse midwives and staff nurses, who are classified as *non-prescribers* because they do not have prescribing authority. This classification illustrates the salience of medication as a defining feature for categorizing primary care staff.

Mental health services were delivered by government health workers trained and supervised by TPO Nepal. Prescribers were trained in a 10-day curriculum roughly divided between basic psychosocial skills and mhGAP content for four disorders (depression, psychosis, alcohol use disorder, and epilepsy). Non-prescribers were trained for 5 days on basic psychosocial support skills, and a subset of these non-prescribers received additional training in manualized psychological interventions for depression and alcohol use disorder (behavioral activation and motivational enhancement therapy, respectively), which had been modified for delivery by people who are not psychiatrists or psychologists in South Asia [31]. For both prescribers and non-prescribers, the standard training was conducted solely by mental health specialists: psychiatrists for mhGAP material and experienced psychosocial counselors for the psychosocial components and manualized psychological interventions.

An alternative training strategy was developed (RESHAPE), in which training was facilitated not only by mental health specialists but also by PWLE and aspirational figures. In RESHAPE, a participatory photography method (PhotoVoice) was used to help PWLE develop recovery narratives that they presented during the training accompanied by photographs they had taken [32,33]. In addition, PCPs who had previously received training on mental health services and who had regularly integrated mental health treatment for their patients were recruited to provide narratives of their experiences. They were termed ‘aspirational figures’ because of the hope that other PCP trainees would aspire to similar commitment to mental health service delivery. A full description of the RESHAPE training curriculum has been published previously [24]. Table 1 presents the contents of the 10-day training for prescribers and 5-day training for non-prescribers, as well as the elements that constitute RESHAPE [27].

Thirty-four health facilities in Chitwan were eligible for participation in the study. The primary care facilities comprised the unit of clustering. Facilities were randomly assigned to either the standard mhGAP training or the RESHAPE adaptation of mhGAP training. All PCPs at a given facility received the training relevant to the arm of randomization, i.e., all prescribers and non-prescribers at a facility were in the same arm (standard mhGAP or RESHAPE). This minimized the risk of contamination across arms. Full details of the cluster design are in the study protocol [22] and details of cluster retention are available in the pilot primary outcome publication [27].

### 2.2. Data Collection and Analysis

#### 2.2.1. Qualitative Data

Semi-structured qualitative interviews were conducted 6 months post-training. Qualitative interview participants were purposively recruited. The sample included a subset of PCPs in mhGAP training, PWLE involved as co-facilitators, and caregivers of PWLE of the training. Caregivers attended the PhotoVoice training with PWLE. Having caregiver engagement in training has been linked to more support and greater participation in training for PWLE [32]. The interview content focused on participants’ reflections on the training and their current practices at the health post in regard to treatment for mental illnesses. Interviews were conducted in Nepali and lasted approximately 90 min and were conducted in the same training location each time. Interviews were first transcribed in Nepali and then translated into English. Data analysis followed a thematic approach and was completed using NVivo 11 [34]. A codebook was developed with each code having its own unique definition and inclusion/exclusion criteria [35]. Three members of the data analysis team (AB, CL, and DG) coded the interviews. Before starting the coding, these three raters completed an inter-rater reliability (IRR) process. They independently coded transcripts, discussed reasons for coding disagreements, and adjusted code definitions and coding strategies to improve agreement. IRR among the three coders reached 0.79 before formal coding was initiated. In total, the data analysis team went through twenty percent of the total transcripts to develop codes until meaning saturation and agreement amongst coders was reached (i.e., reviewing additional transcripts until in the diversity of code meanings was covered [36]). Final coding was completed on all interviews. The first author developed summaries of codes relevant to this manuscript, including attitudes toward psychological treatment and medication, and conducted structured comparisons to identify differences in perceptions and experiences by type of training (standard mhGAP vs. RESHAPE).

#### 2.2.2. Quantitative Data

The health workers participating in mhGAP training in both arms completed assessments of knowledge and attitudes pre-training and at multiple follow-up points as part of a larger suite of quantitative assessments [22]. Here, we focus on specific questions from the Depression Attitudes Questionnaire (DAQ) [37] and the mhGAP Knowledge Assessment adapted for PRIME [38]. The DAQ comprises twenty statements that measure a practitioner’s attitudes towards depression. Specifically, these attitudes can be grouped into four themes: unease with interaction with depressed patients, belief that depressive symptoms are inevitable, acknowledgement and understanding that depression is a clinical disorder, and treatment of depression. The DAQ can be reported as individual item results or as subscale totals [39,40]. For the purpose of these analyses, a subset of these questions allowed us to evaluate attitudes related to this analysis’ objective of attitudes toward psychological vs. pharmacological treatments. Disorder-specific questions about counselling were only asked about depression and alcohol use disorder because these were the two conditions for which a manualized psychological intervention was included. ANOVAs with post-hoc tests were used to compare pre/post scores in continuous outcomes of interest, and chi-squared tests were used to compare changes in the frequency of correct answers or positive attitudes between the first assessment period (pre-training) and a subsequent follow-up (16 months post-training).

## 3. Results

### 3.1. Qualitative Results

#### 3.1.1. Participants and Overview of Themes

For the qualitative interviews, 45 people participated: 3 non-prescribers and 8 prescribers from standard mhGAP training; and 7 non-prescribers and 11 prescribers from RESHAPE training. In addition, 9 PWLE and 7 of their caregivers participated in the qualitative interviews. No aspirational figures participated. 

#### 3.1.2. Attitudes toward Medications and Psychological Treatments Prior to Training

In the qualitative interviews conducted 6 months post-training, participants retrospectively reported that pre-training, they had focused on pharmacological treatment methods for mental illnesses. When asked how to treat patients with mental illness, participants described prescribing a variety of medications. Many expressed the sentiment that medications are a way to quickly get patients back to the functioning levels that they had before their mental health problems started. This would then help them be integrated into the community again:

*“When confronted with people who would complain of their mental health problems, we would try to bring awareness by explaining that mental health problems can be treated in a hospital with medication and such. We told the patients that they would be able to get back to their day-to-day activities just like they were before they had mental health problems.”*

*—#15, RESHAPE prescriber, Male.*

Findings also reflected the stigma that exists towards people with mental illness, as well as those who treat them. A RESHAPE prescriber expressed, *“I used to be scared of clients with mental health issues. I used to think that maybe they will hit me. I had no idea how to deal with such clients”* (#16, male). Other prescribers and non-prescribers expressed similar views and experiences, most commonly not wanting to talk to a patient with mental illness for a long period of time and fear of violent behavior from the patient.

PCPs said that medications somewhat overcame this stigma because the act of prescribing medications for mental illness was consistent with care for physical illnesses. Thus, patients with mental illnesses were not singled out by getting a different form of care. Moreover, because the medications were delivered by PCPs—as opposed to psychiatrists—health workers thought that patients would not feel as stigmatized:

*“If a doctor prescribes those medicines, they stigmatize them as a ‘mad man’s doctor’ (*Nepali: paagal ko daktar*). But they don’t say that to us because we have also dealt with patients with tuberculosis, leprosy, and malaria. We deal with all these patients here and give medications for all these problems, and along with these, we have also started giving medicines for mental illness, and it’s been much easier for us.”*

*—#18, RESHAPE prescriber, Male.*

From the perspective of patients interviewed, taking psychiatric medications is stigmatizing, but taking medications is easier to hide than seeking other types of treatment. For example, a patient could furtively take the medication without family members knowing about it. Prior to the training, family members also had a preference for medication because they could surreptitiously give it to the patient (e.g., mixed in their food) without the patient’s consent or knowledge. In our study, prior to the training, one PWLE with depression was unaware that she was on psychiatric medications because her family would erase the name of the drug on the package before giving it to her. This finding points to the notion that psychotropic medications are not free from stigma. The stigmatization of psychiatric medication was raised by multiple PCPs:

*“Mental health stigma should be reduced. High class people don’t want others to know that they are mentally ill. They simply say that they have been cursed by some supernatural power. When they get cured by traditional healers, they feel as if god has cured them. But, if they take medicines from a health post, people look down upon them and say they had a mental disease and needed medicines to be cured. When we give them these medicines, they think that they are cured by medicines for crazy people.”*

*—#49, RESHAPE prescriber, Male.*

A few providers said that before the training, they believed that mental illnesses were untreatable. Additionally, some health workers acknowledged that they held some stigma towards people with mental illnesses and reported that they dealt with this by quickly getting the patient out of the clinic by writing them a prescription. Hurriedly writing a prescription reduces the time the clinician spends with the patient, and the patient will typically leave satisfied by having a medication.

PCPs also often focused on treating physical symptoms rather than psychological ones. A RESHAPE prescriber described:

*“Firstly, clients with mental issues used to come to us with physical problems such as headaches or fatigue or similar body issues. If a client came in with a headache, we simply prescribed cetamol (acetaminophen) and let them be on their way. We did not dig deeper into their problem.”*

*—#16, RESHAPE, prescriber, Female.*

In some cases, the PCPs reported that prior to training, they typically gave out non-psychiatric medication even if psychiatric symptoms were identified:

*“Sometimes the patients need psychiatric medicine, but they [health care workers] give out other medicines because these kinds of [physical] complaints also came from the clients. As I said earlier, the major learning I received is that now I can identify mental diseases like anxiety or depression, which earlier I would ignore just by giving some headache medicines.”*

*—#14, RESHAPE prescriber, Male.*

#### 3.1.3. Attitudes toward Medications and Psychological Treatment after Training

When asked how the mental health training changed their attitudes, the PCPs described a number of issues. From the qualitative interviews, the knowledge gained in training was associated with more confidence in their ability to diagnose and treat mental health disorders:

*“Interviewer: Now, after the training, what aspects of mental health services are you confident in providing?”*

*“Respondent: After taking the mental health service help, if a client comes, I can differentiate between depression, psychosis, epilepsy, and alcohol withdrawal. Likewise, I can follow up with counseling. They say that for those with mental health issues, counseling can help solve half of their problems.”*

*—#16, RESHAPE prescriber, Female.*

The fact that the training was offered to both prescribers and non-prescribers fostered cooperation between the two groups, and almost all participants expressed that it helped streamline the new focus in treatment methods. For example, a RESHAPE prescriber explained:

*“Both prescribers and non-prescribers are playing good roles. After we see a patient, we send the patient to the non-prescriber for counseling… When the patient comes for follow up visits, we can decide whether the patient needs medicines or only counseling. First, we send them for counseling, and after counseling only, we decide whether the medicines are required or not.”*

*—#18, RESHAPE, prescriber, Male.*

Within the psychosocial training components, mental health and illness were framed with the local psychological concept of the heart-mind (Nepali: *man*, 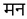), which is the organ of memory and emotion. Psychological distress in the heart-mind is seen as commonplace, thus not highly stigmatized. By further understanding this term, participants came to recognize the necessity of talking to patients with mental illnesses by listening to their stories so that the healthcare worker can get to the root of the patient’s problem, as reported by both prescribers and non-prescribers:

*“Before, when such patients with problems related to heart-mind used to come, we used to refer them directly, but now we look after the patients. We prescribe medicine to them, and we also provide them counseling so it has been very easy”*

*—#12 RESHAPE prescriber, Male.*

*“In the case of heart-mind problems, I think counseling is very helpful. However, as cases differ, the use of medicine might also be required. I feel that the counseling is really important as through psychosocial counseling, we can reach the heart-mind of that person and know things, which might in turn be helpful in curing them”*

*—#1, RESHAPE non-prescriber, Female.*

The RESHAPE component of training was woven into the psychosocial and mhGAP components. Participants described that the addition of PWLEs as co-facilitators in the training humanized people with mental health disorders for them. Being able to hear the stories from PWLEs, see pictures of their day-to-day lives, and openly interact with them allowed the participants to have a tangible recovery story to refer back to and think about. The in-person PWLE recovery testimonials mentioned counseling as something that helped their recovery, which may have added to the PCPs’ changed perception that counseling is a valid way to treat mental illness. Counseling and listening were mentioned in a positive manner by trainee participants from both study arms:

*“We had no idea about the counseling before. We used to ask them a few questions and then referred them to doctors or give them suggestion to meet them. But now, we try to understand their problems. Counseling is also as important as medication. If both the counseling and medication is provided to the patient, then it will help them to recover soon. There will be changes in their lives.”*

*—#34. RESHAPE prescriber.*

In addition to PWLEs, the RESHAPE training included aspirational figures as co-facilitators. These aspirational figures were previously trained healthcare workers who successfully treated people living with mental illness. These figures were selected by supervisors as good role models for current trainees.

*“I: What benefits do you think there were because of [aspirational figures’] involvement in the training?”*

*“R: I mentioned about it before too. It helped us in part of counseling too. When we listened to what the health workers said, it made us feel that we can help [people with mental illness] for their treatment in our health post too. We can help them achieve the life they had before having this illness. It motivated us.”*

*—#2, RESHAPE non-prescriber, Female.*

#### 3.1.4. Structural Factors

Participants mentioned structural challenges in implementing mental health services. The three main structural factors that affected provision of mental health services were supply of medications at the health facility, lack of human resources, and inadequate infrastructure. Through the PRIME program, medications have been made free, yet they were not always in stock at the health facility. Providers mentioned that this was an issue they encountered at all time points and said that there were times when they knew they should give a certain medication to a patient, but it was unavailable at their health facility.

**Figure 1:**
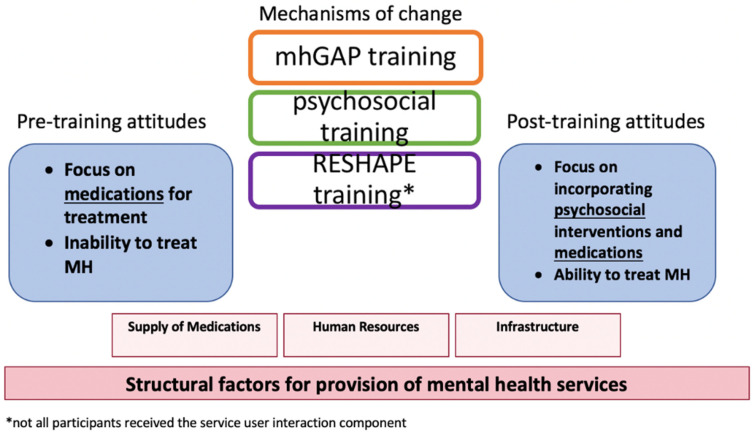
Overview of key components of training and underlying structural factors. Abbreviations: MH, mental health; mhGAP, mental health Gap Action Programme; RESHAPE, Reducing Stigma among HealthcAre ProvidErs.

Table 2 shows how PCPs endorsed subthemes related to attitudes before and after training, presented in order of descending frequency. In regards to retrospective reflections on pre-training attitudes, stigma was the most endorsed subtheme; in reports of post-training attitudes, support for counseling was the most commonly endorsed subtheme. There were no identified differences between the training arms or between non-prescribers and prescribers. Figure 1 illustrates the changes from pre- to post-training attitudes, the possible mechanisms of change, and the relationship to underlying structural factors.

### 3.2. Quantitative Results

A total of 205 PCPs took part in the quantitative assessments: 110 prescribers and 95 non-prescribers. Demographic details of participants at baseline are provided in Table 3.

**Figure 2:**
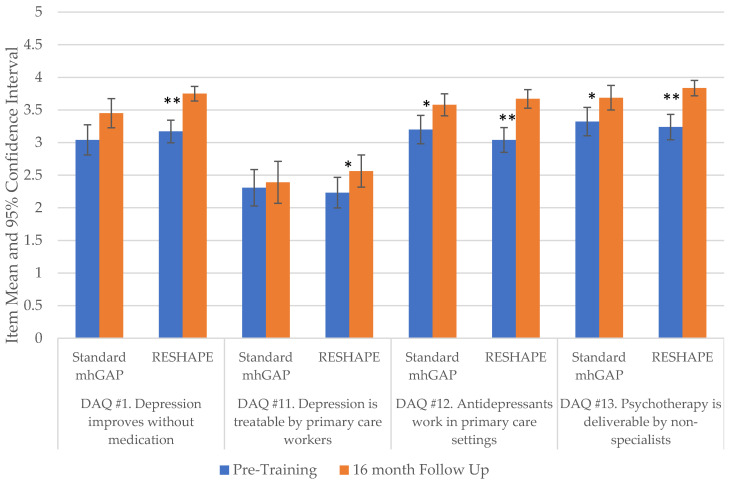
Changes between pre-training and 16 month follow-up among PCPs on specific questions pertaining to treatment of depression from the Depression Attitudes Questionnaire (DAQ). * *p* < 0.01, ** *p* < 0.001; Abbreviations: mhGAP, mental health Gap Action Programme; PCP, primary care provider; RESHAPE, Reducing Stigma among HealthcAre ProvidErs.

ANOVA testing was used to compare changes over time in attitudes towards depression. Among standard mhGAP trainees, there were significant differences between baseline and the 16 month follow-up on three out of four Depression Attitudes Questionnaire (DAQ) questions: standard mhGAP DAQ #1 “Depression can improve without medication” (*F* = 8.68, *p* < *0*.001); DAQ #12 “Antidepressants work in primary care” (*F* = 1.87, *p* = *0*.049)); and DAQ #13 “Psychotherapy is deliverable by non-specialists” (*F* = 4.78, *p* = *0*.003), see Figure 2 and Supplemental Table S1. However, there was no significant difference found on DAQ #11 “Depression is treatable by primary care workers” (*F* = 2.17, *p* = *0*.092). For the RESHAPE study arm, we found significant increased endorsement across all four questions: DAQ #1 (*F* = 9.83, *p* < *0*.001), DAQ #11 (*F* = 4.40, *p* = *0*.005), DAQ #12 (*F* = 9.04, *p* < *0*.001), and DAQ #13 (*F* = 11.64, *p* < *0*.001).

**Figure 3:**
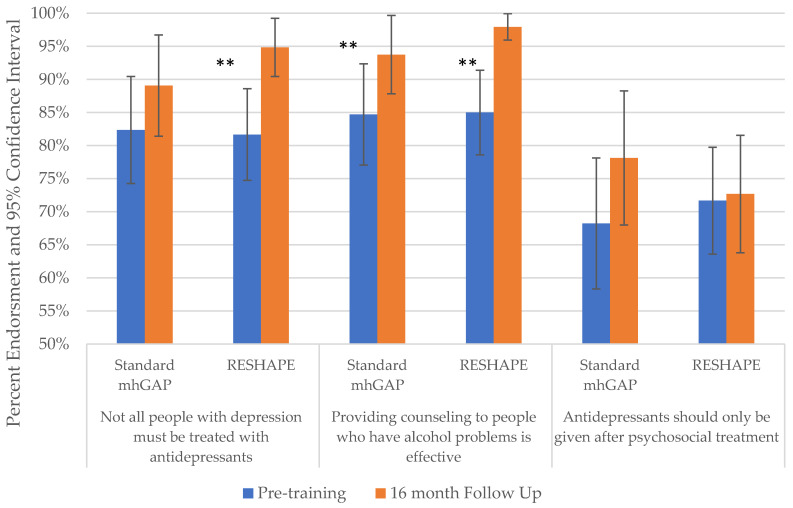
Changes between pre-training and 16 month follow-up among PCPs on specific questions pertaining to medication and counseling from the PRIME mhGAP knowledge test. Abbreviations: mhGAP, mental health Gap Action Programme; PCP, primary care provider; PRIME, Program for Improving Mental Health Care; RESHAPE, Reducing Stigma among HealthcAre ProvidErs. ** *p* < 0.001.

Chi-squared analyses were used to compare changes in frequency of endorsement of mhGAP knowledge items from pre-training to 16 month follow-up. The item “Antidepressants should only be given after psychosocial treatment” did not show statistically significant changes for either standard mhGAP (*χ*^2^ = 2.48, *p* = 0.480), (*χ*^2^ = 5.49, *p* = 0.139) or RESHAPE trainees (*χ*^2^ = 3.33, *p* = 0.344), (*χ*^2^ = 1.37, *p* = 0.714), see Figure 3 and Supplemental Table S2. However, endorsement of “Providing counseling to people who have alcohol problems is effective” increased significantly for both standard mhGAP (*χ*^2^ = 10.01, *p* = 0.019) and RESHAPE trainees (*χ*^2^ = 26.201, *p* < 0.001). Among standard mhGAP trainees, “All people with depression should be treated by antidepressants” did not show statistically significant changes (*χ*^2^ = 7.04, *p* = 0.071), but it did for RESHAPE trainees (*χ*^2^ = 17.62, *p* < 0.001).

## 4. Discussion

In qualitative interviews six-months after mental health training, PCPs described a shift in their perceptions about pharmacological and psychological treatments for mental illnesses. These shifts were described by non-prescribers and prescribers in both study arms. When reflecting on their attitudes and behaviors before the training, PCPs in both arms stated they were more willing and likely to treat people with mental illnesses using medications. Many of the stigmatizing attitudes that characterized PCP attitudes prior to training are comparable to perspectives of PCPs around the world, as recently demonstrated in qualitative research in seven countries representing both HIC and LMIC settings, with the latter including Lebanon, Tunisia, India, and Nepal [41].

After the training, there was increased support for and endorsement of the effectiveness of counseling as a treatment for mental illnesses. Additionally, both prescribers and non-prescribers expressed that they felt more comfortable talking to mental health patients and asking questions about symptoms of mental illnesses. For non-prescribers, they felt empowered by gaining knowledge to directly help patients with mental illnesses rather than only referring such patients to prescribers as they had done in the past. For evidence-based psychological treatments to be successful in LMIC settings, it is necessary to have buy-in from all levels of healthcare providers, along with recognizing the value of the treatment [42]. Thus, it is a positive sign that the non-prescribers have found the role of counseling patients with mental illnesses as one they can fulfill. However, in other LMIC settings, some psychosocial counselors have not felt comfortable presenting psychological treatments and have often reframed them in a biomedical and psychiatric lens [43].

There was not a clear difference between standard mhGAP trainees (who received mhGAP and psychosocial training components) and RESHAPE trainees (who received an additional stigma-reduction component involving PWLEs and aspirational figures) in regard to their qualitative endorsement of counseling based on interviews held 6 months post-training. This may be because of the training modality included 5 days of psychosocial support training alongside 5 days of mhGAP. There may have been additional benefit from the training arm in which PWLEs provided recovery stories and co-facilitated sections of the training. Specifically, recovery stories showed that counseling is a viable treatment option. Another qualitative analysis attached to the RESHAPE study corroborates the benefit of PWLEs’ recovery stories as a method to reduce stigma [44]. The same study points out the need to reduce self-stigma and the lack of PWLE involvement within governmental initiatives.

There were differences between training arms in quantitative assessments of stigma towards people with mental illness, as well as questions asking if mental illness can be treated without medications in a primary care setting. Specifically, RESHAPE participants showed a significant increase in endorsing that depression is treatable by primary care workers and that depression does not need to always be treated with antidepressants. Similar attitudinal and knowledge shifts in relation to the psychosocial context of depression have been found in Nigeria [5]. In Tunisia, similar shifts were seen in primary care physicians being more comfortable engaging with potential mental health patients, along with an increased knowledge of alternative treatments beyond pharmacology for mental illnesses [9].

It is just as important to consider how PWLEs think about perspectives of mental health treatments for mental illness. In our study, both PWLEs and PCPs sometimes felt that it was less stigmatizing to take psychotropic medications than engage in counseling. This sentiment is echoed in HICs as well: in the United States, it was found that stigma affected the acceptability of treatment modalities for depression among Hispanic and African American populations, with herbal remedies being the least stigmatizing compared to prescription medication or counseling [45]. In Nepal, other research has suggested that people experiencing mental illness are more likely to pursue traditional healing as the first line of care, which may be due to less stigma with traditional healers and also the lack of a formal distinction between physical and psychological distress when seeing these healers [7,18,19,20,21,46].

There may also be hesitation from PWLEs to seek counseling until a trustworthy bond is made with the counselor [47]. However, in prior studies of counseling in Nepal, 91% of patients were satisfied with the counseling services offered, and 73% felt that it effectively addressed most of their needs [47]. Other components of the PRIME study in Nepal demonstrated that referral to a community psychosocial counselor has the added benefit for depression, compared to treatment predominantly entailing medication from PCPs [31]. In-person testimonials from PWLEs in RESHAPE may encourage PCPs to listen to and spend more time with their patients to build rapport. Notably, the personal testimonials appear to only be effective in changing attitudes across different types of mental illnesses when performed in-person; in an RCT performed in Nepal with video-taped testimonials from PWLEs, there were mixed results, with stigma worsening for video testimonials of people living with psychosis [48].

Structural barriers such as lack of counseling rooms (alluding to the fact that confidentiality may not be assured), staff turnover leading to difficulty in maintaining trustworthy bonds, and work burden of PCPs may hinder PWLE and health workers’ attitudes towards psychological treatments. Therefore, efforts are also needed to mitigate structural stigma [49].

One of the potential challenges in promoting psychological mental health services is how these become mapped onto existing health system cadres. For example, in Nepal, there is the existing division of prescribers and non-prescribers in primary care. Because of time availability for service delivery, it was found to be more feasible to train non-prescribers in the manualized psychological interventions. Although we piloted training prescribers on motivational enhancement therapy, they did not have adequate time to participate in training and to deliver the therapy in routine care. Therefore, by the fact that non-prescribers have a lower level of training and status in the health system, only having non-prescribers performing manualized psychological interventions may implicitly send a message that these are lower forms of care compared to medication. However, a positive outcome of the qualitative findings in the current study was that both prescribers and non-prescribers described more collaboration as a result of the training. This was one of the key themes raised by many of the PCPs, which suggests that the training encourages a collaborative approach to psychological services including encouragement for counselling from prescribers. Another positive factor is that WHO has developed brief training in foundational skills and associated competency evaluations that can be used with health workers and others even if they do not complete full manualized intervention training [50,51].

There are a number of limitations to our study. We do not know if the attitudinal shifts described in interviews and surveys are borne out in PCPs’ behavior. We are unable to pinpoint exactly what element of the training is the most impactful in making this shift in attitudes. Because interviews were all completed following the training, it may be hard for the participants to remember exact aspects of the training or articulate how those aspects affected their attitudes and behaviors. The scales also used to measure these changes were not all adapted for our setting. Interviews were all conducted by TPO Nepal staff members, so some of the answers may be biased by presumed social desirability of endorsing psychological services. Another limitation was the lack of qualitative information from aspirational figures, which would have been helpful to gain their perspective on what influenced PCPs’ attitudes toward psychological care. Questions about counseling and attitudes were only asked about depression and alcohol use disorder, due to psychological interventions only being taught for depression and alcohol use disorder. Ideally a prospective study would capture both attitudes and changes in clinical behaviors over time. A prospective study would also have allowed for observations of the type of treatment used for mental health patients to be analyzed. Further studies can incorporate counseling and attitude questions for other mental illnesses.

Subsequent steps in the research area will be to explore how attitudes are associated with observer evaluations of clinical skills [52,53]. We must also examine how providers use medications and counseling techniques post-training in primary care settings for mental health services. It would be of interest to formally study what the patients’ preferences are between pharmacological and psychological treatments in LMIC settings.

## 5. Conclusions

These findings suggest that including psychological treatment knowledge and skills and integrating stigma-reduction components into the mhGAP training package may be useful in encouraging psychological interventions in primary care settings. Encouraging social contact of PWLEs with primary care health workers and providing in-person testimonials on recovery experiences by PWLEs may help reduce stigma and increase the uptake of psychological services. In addition, training and supervision approaches are needed that highlight the effectiveness of psychological support integrated into pharmacological programs. Our findings suggest that collaboration with PWLEs and aspirational figures’ advocacy for psychological treatments increase the likelihood that primary care health workers recommend psychological treatments. This should be combined with assuring appropriate structural conditions, such as confidential spaces for psychological treatment, in all primary care facilities. Taken together, these efforts could increase the likelihood of primary care health workers endorsing and facilitating delivery of psychological services to patients even in low-resource settings.

## Figures and Tables

**Table 1:** Curriculum for primary care providers in mhGAP training.

	**Day 1 ^a^**	**Day 2 ^a^**	**Day 3 ^a^**	**Day 4 ^a^**	**Day 5**
**Morning session**	Introduction to training Objectives	* Aspirational figure testimonial, and common myths Introduction to psychosocial problems, causes and symptoms	* In-person PWLE recovery stories with Q&A Communication skills	Psychosocial skills training	Introduction to psychiatric diagnoses and mhGAP-IG
**Afternoon session**	Intro to mental health and psychosocial concept Pre-tests	Introduction to psychosocial support	Communication skills	Psychosocial skills training * Reducing Stigma	mhGAP curriculum
	**Day 6**	**Day 7**	**Day 8**	**Day 9**	**Day 10 ^a^**
**Morning session**	Psychiatric history taking skills Epilepsy assessment, diagnosis	Depression and suicide assessment and diagnosis	Psychosis and bipolar assessment and diagnosis	Alcohol and drug use disorder assessment diagnosis and management * In-person PWLE recovery story with Q&A	Documentation and supervision
**Afternoon session**	* In-person PWLE recovery story with Q&A Clinical patient evaluation	* In-person PWLE recovery story with Q&A Clinical patient evaluation	* In-person PWLE recovery story with Q&A Clinical patient evaluation	* Challenges and barriers: collaborative problem solving	Post-test

Abbreviations: mhGAP-IG, mental health Gap Action Programme-Implementation Guide; PWLE, people with lived experience of mental illness; Q&A, question and answer session; ^a^ Non-prescriber content was for Days 1–4 and Day 10 (total 5) and was conducted separately from prescribers. * Specific to RESHAPE training.

**Table 2:** Endorsement of subthemes by primary care providers in RESHAPE arm.

Theme	Sub-Theme *	Description
PCPs’ reported attitudes prior to mental health training	(1) Stigma towards MH medications	Stigma exists towards those who take and those who prescribe medications for mental illnesses
	(2) Focus on physical symptoms and treatment of those	PCPs focused on treating somatic symptoms rather than the underlying psychological ones and thus prescribed medications to treat the physical symptoms
	(3) Easy, cheap, and quick mode of treatment	Medications are perceived by healthcare workers to be easy to prescribe and a cost-effective method of treatment
	(4) Medications are the quick way back to normal	Perception that medications equate to a quick and easy way to get a MH patient back to doing daily activities
PCP’s reported learning after training	(1) Endorsement of counseling	Healthcare workers became aware of the technique of counseling and endorsed the efficacy of counseling as a treatment for MH patients
	(2) Listen and spend time with patients to understand their problems	Participants expressed the necessity to listen to patients to understand the root of the symptoms that brought he/she to the health post
	(3) Increased knowledge of mental illness	Through the training, PCPs expressed that their knowledge about MH disorders relevant to their geographic area had increased
	(4) Increased cooperation between prescribers and non-prescribers streamlined services	With the training, there has been an increase in the cooperation between the prescribers and non-prescribers helping serve the patients better. Non-prescribers perform counseling and can spend time with patients, then prescribers provide prescriptions.
Attributions for changes in knowledge and attitudes	(1) Psychosocial content	Techniques for how to interact with people with MH disorders
	(2) mhGAP content	General knowledge of mental health disorders
	(3) RESHAPE content	Addition of in-person PWLEs’ testimonials

* Subthemes are presented in order of descending frequency in qualitative data. Abbreviations: MH, mental health; mhGAP, mental health Gap Action Programme; PCP, primary care provider; PWLE, people with lived experience of mental illness; RESHAPE, Reducing Stigma among HealthcAre ProvidErs.

**Table 3:** Baseline demographics of primary care providers in the study.

Baseline Demographics	N (%)
*Gender*	
Female	96 (46.8%)
Male	109 (53.2%)
*Age*	
21–29 years	74 (36.1%)
30–39 years	56 (27.3%)
40–49 years	52 (25.4%)
50+ years	23 (11.2%)
*Caste/Ethnicity*	
High caste groups	144 (70.2%)
Lower caste groups and ethnic minorities	61 (29.8%)
*Type of primary care provider*	
Non-prescriber ^A^	95 (46.3%)
Prescriber ^B^	110 (53.7%)
*Years working in healthcare services* ^C^	
<1 year	10 (4.9%)
1–5 years	62 (30.2%)
6–10 years	25 (12.2%)
>10 years	107 (52.2%)

^A^ Primary care provider without prescribing rights, e.g., auxiliary nurse midwife. ^B^ Primary care provider with prescribing rights, e.g., health assistant. ^C^ Missing data on one participant.

## Data Availability

Data are available from the authors upon reasonable request.

## Supplementary Materials

The following supporting information can be downloaded at: https://www.mdpi.com/article/10.3390/ijerph19042149/s1, Table S1: Comparison of Depression Attitudes Questionnaire (DAQ) results between standard mhGAP training and RESHAPE; Table S2: Comparison of mhGAP attitudes questions between mhGAP training and RESHAPE.
