# Supplementary material for: Treatment Preferences for Pharmacological versus Psychological Interventions among Primary Care Providers in Nepal: Mixed Methods Analysis of a Pilot Cluster Randomized Controlled Trial
Source: Int J Environ Res Public Health. 2022 Feb 14;19(4):2149. doi: 10.3390/ijerph19042149 (PMC8871897; doi:10.3390/ijerph19042149)
Supplement: Supplementary file 1 [file ijerph-19-02149-s001.zip › ijerph-1549461-supplementary.pdf]

Table S1. Comparison of Depression Attitudes Questionnaire (DAQ) results between standard mhGAP training and RESHAPE

	Standard mhGAP		RESHAPE	
DAQ Questions	F Statistic	p-value	F Statistics	p-value
DAQ#1. Depression improves without medication	8.68	0.001	9.83	<0.001
DAQ#11. Depression is treatment by primary care workers	2.17	0.092	4.40	0.005
DAQ#12. Antidepressants work in primary care settings	1.87	0.049	9.04	<0.001
DAQ#13. Psychotherapy is deliverable by non-specialists	4.78	0.003	11.64	<0.001

Table S2. Comparison of mhGAP attitudes questions between mhGAP training and RESHAPE

	Standard mhGAP		RESHAPE	
mhGAP Knowledge Question	χ^2	p-value	χ^2	p-value
Not all people with depression must be treated with antidepressants	7.04	0.071	17.62	<0.001
Providing counseling to people who have alcohol problems is effective	10.01	0.019	26.201	<0.001
Antidepressants should only be given after psychosocial treatment	2.48	0.480	5.49	0.139
